# A Fundamental Step in IPM on Grapevine: Evaluating the Side Effects of Pesticides on Predatory Mites

**DOI:** 10.3390/insects6040847

**Published:** 2015-10-09

**Authors:** Alberto Pozzebon, Paola Tirello, Renzo Moret, Marco Pederiva, Carlo Duso

**Affiliations:** Department of Agronomy, Food, Animals, Natural Resources and Environment (DAFNAE), University of Padova, Viale dell’Università 16, Legnaro-Padova 35020, Italy; E-Mails: alberto.pozzebon@unipd.it (A.P.); paola.tirello@unipd.it (P.T.); renzo_moret@alice.it (R.M.); commerciale@proseccoserre.it (M.P.)

**Keywords:** side-effects, chemical treatments, *Vitis vinifera*, Phytoseiidae, *Typhlodromus pyri*, *Amblyseius andersoni*

## Abstract

Knowledge on side effects of pesticides on non-target beneficial arthropods is a key point in Integrated Pest Management (IPM). Here we present the results of four experiments conducted in vineyards where the effects of chlorpyrifos, thiamethoxam, indoxacarb, flufenoxuron, and tebufenozide were evaluated on the generalist predatory mites *Typhlodromus pyri* Scheuten and *Amblyseius andersoni* (Chant), key biocontrol agents of herbivorous mites on grapevines. Results show that indoxacarb and tebufenozide had a low impact on the predatory mites considered here, while a significant impact was observed for chlorpyrifos, flufenoxuron, and thiamethoxam. The information obtained here should be considered in the design of IPM strategies on grapevine.

## 1. Introduction

Knowledge of the impact of pesticides on beneficial arthropods is critical in Integrated Pest Management (IPM). The authorization of a new pesticide in Europe is subjected to the evaluation of its effects on non-target organisms, including a number of beneficials. These evaluations can be made at different levels (e.g., laboratory, semi-field, and field) obtaining complementary information [1,2]. Most of the data in the specific literature come from laboratory and semi-field studies as they are less time-consuming and expensive than field tests. However, field tests are useful for evaluation of the pesticides’ impact in realistic use conditions, especially for pesticides having a significant impact in laboratory or semi-field studies [3,4,5]. Predatory mites belonging to the Phytoseiidae family, particularly generalists *sensu* McMurtry and Croft [6], have been widely considered in field evaluations of pesticide side-effects (e.g., [2,7,8,9]). Generalist predatory mites can persist on perennial crops when prey is scarce by feeding on alternative foods and prevent herbivore mite outbreaks for a long time [10,11,12]. Thus, the assessment of pesticide impact on them can be performed excluding potential effects mediated by their prey [13]. At the same time, plant-pathogens, especially those involved in fungal diseases, can represent alternative or supplemental food resources for various predatory mites and their presence can alter the outcome of trials aimed at assessing the impact of pesticides [14,15,16]. Field experiments should therefore be conducted where symptoms caused by fungal diseases are negligible to low.

There is an abundant literature on the side-effects of pesticides on predatory mites but the variability of environmental factors, experimental methods, target species, and pesticide formulates makes comparison of these data difficult. An attempt to summarize the results conducted using standardized methods is reported in the IOBC Pesticide Side Effect Database published by the International Organisation for Biological and Integrated Control/West Palearctic Regional Section (IOBC/WPRS) [17]. Pesticides are classified according their overall effects (Classes 1–4, from harmless to harmful), providing selectivity rates to people involved in IPM. When studied, lethal and sub-lethal effects are combined and, thus, cited publications should be examined by those interested in investigating these aspects. Nevertheless, results presented in reports used in regulatory procedures for pesticides’ use authorization are often reported giving useful information to IPM practitioners.

In this paper we summarize the results of a number of experiments conducted in vineyards to evaluate the effects of a number of insecticides on the generalist predatory mites *Typhlodromus pyri* Scheuten and *Amblyseius andersoni* (Chant). These species are key biocontrol agents of herbivorous mites [11,18,19,20,21,22,23]. The choice of insecticides was based on their importance in pest control strategies in viticulture. For some insecticides, it was possible to compare our results with those reported in the IOBC Pesticide Side Effect Database. For other pesticides, records could integrate gaps in the above-mentioned database.

## 2. Experimental Section

The effects of insecticides on predatory mites were investigated in four vineyards located in the Veneto region (Northeastern Italy), two comprising cultivar Glera grapevine (experiments 1 and 2; Miane and San Pietro di Feletto, respectively, province of Treviso; Table 1), one with cultivar Merlot (experiment 3; Spresiano, province of Treviso; Table 1), and one with cultivar Cabernet Sauvignon (experiment 4; Spresiano, province of Treviso; Table 1). These vineyards were inhabited by predatory mites (*T*. *pyri* and *A*. *andersoni*) that are considered key biocontrol agents of herbivore mites as well as target beneficials in the evaluation of pesticide side effects. The impact of insecticides was compared in four field experiments where an untreated control treatment was also included (Table 1). Experimental design was a randomized block with four replicates per treatment. Timing of applications was determined by the mode of action of different insecticides and the phenology of main target pests for each vineyard. In particular, from preliminary observations, *Empoasca vitis* (Goethe) (Hemiptera Cicadellidae) was the target pest in experiments 1 and 2, while *Lobesia botrana* (Denis and Schiffermüller) (Lepidoptera Tortricidae) was the target pest in experiments 3 and 4 (Table 1). Preliminary observations revealed that *T*. *pyri* was the dominant species in the vineyards of experiments 1 and 2, while *A*. *andersoni* was dominant in the vineyards of experiments 3 and 4. Regarding insecticide use in previous years, all these vineyards had been treated with organophosphates, carbamates, and chitin-inhibitors. Therefore, the dominance of these predatory mite species was likely be due to their adaptation to specific environmental conditions rather than pesticide history: experimental sites 1 and 2 were located in hilly areas whereas experimental sites 3 and 4 were in lowland areas. The experiments were performed in mid-summer and the climatic conditions were typical for the area where the experimental sites were located. Samplings were conducted before and approximately every week after insecticide applications for about one month. A total of 100 leaves per treatment (25 leaves per replicate) were removed and transferred to the laboratory where predatory mite individuals were counted under a dissecting microscope; a number of females were mounted on slides, in Hoyer’s medium, and identified under a phase contrast microscope using current keys [24]. Data were analyzed with a restricted maximum likelihood (REML) repeated measures model. Mite densities were considered as response variables with repeated measures made at different times, *i*.*e*., sampling dates. Using an F test (α = 0.05), we evaluated the effect of insecticide application, time, and their interaction. Contrasts (α = 0.05) were designed for pairwise comparison between treatments before and after insecticide applications. Degrees of freedom were estimated using the Kenward-Roger method [25]. According to Akaike’s Information Criterion, first-order autoregressive was chosen as best fitting covariance structure for correlating different sampling dates [25]. Data were checked for analysis assumptions before analyses and log(x + 1) transformation was applied when necessary. The effect (E) of insecticides was estimated using the Henderson and Tilton’s formula [26].

**Table 1:** Experimental design and insecticides applied in the four experiments.

Treatments	Active Ingredients	Commercial Products (a.i. Concentration)	Dose	Timing of Application (Days from First Application)
*Experiment 1*				
control	-		-	-
chlorpyrifos	chlorpyrifos	Dursban 75 WG (75%)	70 g/hL	0 days
thiamethoxam	thiamethoxam	Actara 25 WG (25%)	25 g/hL	0 days
indoxacarb	indoxacarb	Steward EC (30%)	15 g/hL	0 days
*Experiment 2*				
control	-		-	-
chlorpyrifos	chlorpyrifos	Dursban 75 WG (75%)	70 g/hL	0 days
thiamethoxam	thiamethoxam	Actara 25 WG (25%)	25 g/hL	0 days
flufenoxuron	flufenoxuron	Cascade 50 DC (4.7%)	100 mL/hL	0 days
*Experiment 3*				
control	-		-	-
chlorpyrifos	chlorpyrifos	Dursban 75 WG (75%)	70 g/hL	10 days
flufenoxuron	flufenoxuron	Cascade 50 DC (4.7%)	100 mL/hL	0 days
*Experiment 4*				
control	-		-	-
chlorpyrifos	chlorpyrifos	Dursban 75 WG (75%)	70 g/hL	10 days
flufenoxuron	flufenoxuron	Cascade 50 DC (4.7%)	150 mL/hL	0 days
indoxacarb	indoxacarb	Steward EC (30%)	15 g/hL	4 days
tebufenozide	tebufenozide	Mimic (23%)	60 mL/hL	0 days

## 3. Results

### 3.1. Experiment 1

**Figure 1:**
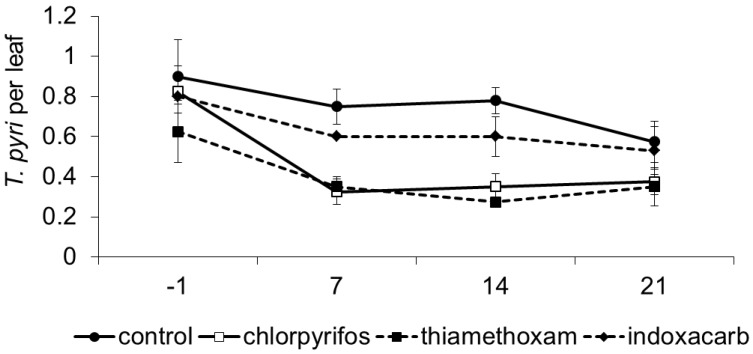
Population dynamics of *Typhlodromus pyri* (mean ± std. err.) observed in different treatments during experiment 1. On the X-axis, days after insecticide application are reported.

*Typhlodromus pyri* was the dominant species in this vineyard, and its density was influenced by treatment and time, while their interaction was not significant (F_3, 11.5_ = 11.70; *p* = 0.001; F_3, 26.7_ = 8.67; *p* < 0.001; F_9, 27.7_ = 1.05; *p* = 0.427; respectively; Figure 1). The occurrence of herbivorous mites (e.g., tetranychids and eriophyids) was negligible. Prior to insecticide applications, *T*. *pyri* densities were similar among plots (F_3, 42.6_ = 1.35; *p* = 0.269). After applications, *T*. *pyri* densities were lower with thiamethoxam and chlorpyrifos as compared to the untreated control (F_1, 13.8_ = 25.35; *p* < 0.001; E = 31.45; F_1, 13.8_ = 21.72; *p* < 0.001; E = 44.21; respectively). No effects were observed for indoxacarb (F_1, 13.8_ = 2.24; *p* = 0.157; E = 6.58; respectively). Among insecticide treatments, predatory mite densities were lower with thiamethoxam and chlorpyrifos than indoxacarb (F_1, 13.8_ = 10.02; *p* = 0.007; F_1, 13.8_ = 7.94; *p* = 0.013; respectively). No differences were observed between chlorpyrifos and thiamethoxam (F_1, 13.8_ = 0.14; *p* = 0.71).

### 3.2. Experiment 2

**Figure 2:**
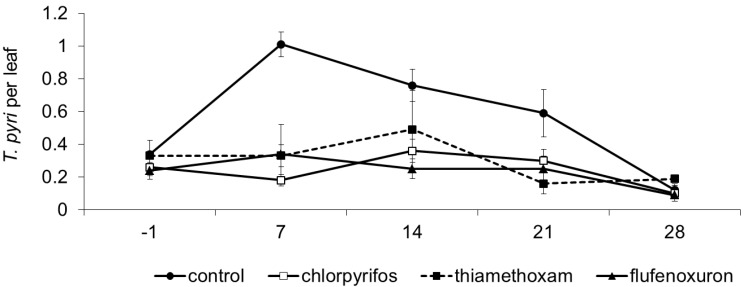
Population dynamics of *Typhlodromus pyri* (mean ± std. err.) observed in different treatments during experiment 2. On the X-axis, days after insecticide application are reported.

*Typhlodromus pyri* was dominant among predatory mites in this vineyard. Its abundance was influenced by treatment, time, and their interaction (F_3, 24.3_ = 9.61; *p* < 0.001; F_4, 46.3_ = 7.72; *p* < 0.001; F_12, 46.7_ = 2.40; *p* = 0.016; respectively; Figure 2). Herbivorous mite populations occurred at negligible levels. In pre-treatment observations, *T*. *pyri* densities were similar among plots (F_3, 57_ = 0.35; *P* = 0.789). Following insecticide applications, *T*. *pyri* numbers were lower where insecticides were applied as compared to the control (thiamethoxam: F_1, 28.1_ = 15.56; *p* < 0.001; E = 27.20; flufenoxuron: F_1, 28.1_ = 22.29; *p* < 0.001; E = 34.85; chlorpyrifos: F_1, 28.1_ = 22.94; *p* < 0.001; E = 34.82). No differences emerged among plots with insecticide applications (thiamethoxam *vs*. flufenoxuron: F_1, 28.1_ = 0.73; *p* = 0.402; thiamethoxam *vs*. chlorpyrifos: F_1, 28.1_ = 0.51; *p* = 0.479; chlorpyrifos *vs*. flufenoxuron: F_1, 28.1_ = 0.02; *p* = 0.893).

### 3.3. Experiment 3

**Figure 3:**
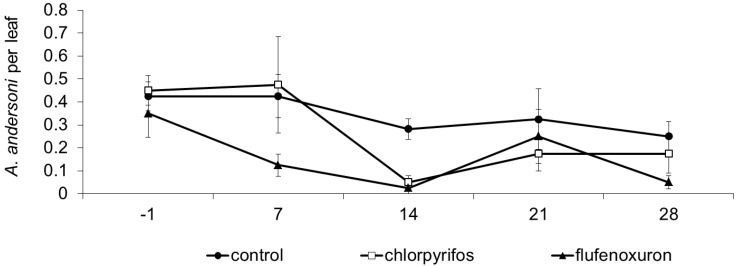
Population dynamics of *Amblyseius andersoni* (mean ± std. err.) observed in different treatments during experiment 3. On the X-axis, days after insecticide application are reported.

In this experiment, the predatory mite *Amblyseius andersoni* was commonly observed on grape leaves. Its population dynamics were influenced by treatment and time, while no effect was found of the interaction “treatment × time” (F_2, 17.9_ = 5.94; *p* = 0.011; F_4, 34.2_ = 5.93; *p* = 0.001; F_8, 34.9_ =1.02; *p* = 0.436; respectively; Figure 3). The occurrence of herbivorous mites was very low. Prior to insecticide application, no differences were observed among treatments (F_2, 44.8_ = 0.37; *p* = 0.691). Later, predatory mite numbers were higher in the control than in flufenoxuron and chlorpyrifos plots (flufenoxuron: F_1, 19.3_ = 12.50; *p* = 0.002; E = 58.95; chlorpyrifos: F_1, 22_ = 5.01; *p* = 0.003; E = 55.43). No differences emerged between the insecticide-treated plots (F_1, 22_ = 0.20; *p* = 0.656).

### 3.4. Experiment 4

**Figure 4:**
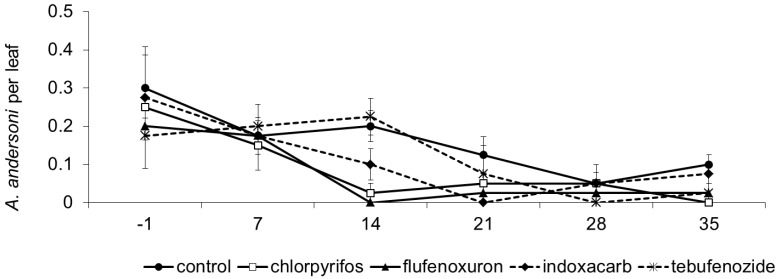
Population dynamics of *Amblyseius andersoni* (mean ± std. err.) observed in different treatments during experiment 4. On the X-axis, days after insecticide application are reported.

In this vineyard *A*. *andersoni* was dominant among predatory mites. Its abundance was influenced by treatment and time, while no effect of their interaction was found (F_4, 35.7_ = 3.36; *p* = 0.012; F_5, 65_ = 15.64; *p* < 0.001; F_20, 68.4_ = 0.97; *p* = 0.510; respectively; Figure 4). The occurrence of herbivorous mites was negligible. Before insecticide application, the abundance of *A*. *andersoni* was similar among plots (F_4, 87_ = 0.91; *p* = 0.462). After insecticide applications, lower predatory mite numbers were found in flufenoxuron and chlorpyrifos than in control plots (flufenoxuron: F_1, 37.5_ = 9.46; *p* = 0.004; E = 41.50; chlorpyrifos: F_1, 40.5_ = 8.58; *p* = 0.006; E = 55.52). No significant effects were observed for indoxacarb and tebufenozide (F_1, 37.5_ = 3.50; *p* = 0.069; E = 29.09; F_1, 37.5_ = 1.13; *p* = 0.295; E = −6.89; respectively). However, it should be noted that predatory mite population densities decreased to zero after 21 days from indoxacarb application and increased thereafter (Figure 4). No differences were observed among insecticides (flufenoxuron *vs*. chlorpyrifos: F_1, 40.5_ = 0.19; *p* = 0.664; flufenoxuron *vs*. tebufenozide: F_1, 37.5_ = 4.06; *p* = 0.051; flufenoxuron *vs*. indoxacarb: F_1, 37.5_ = 1.45; *p* = 0.235; chlorpyrifos *vs*. tebufenozide: F_1, 40.5_ = 2.45; *p* = 0.125; chlorpyrifos *vs*. indoxacarb: F_1, 40.5_ = 0.70; *p* = 0.408; tebufenozide *vs*. indoxacarb: F_1, 37.5_ = 0.66; *p* = 0.423).

## 4. Discussion and Conclusions

The statistical analyses revealed a significant impact of insecticides with E-values exceeding 27%, with the exception of indoxacarb in the fourth experiment (E = 29.1%). In the traditional IOBC classification [2], pesticides that have an overall effect (E) lower than 25% for field trials and 30% for laboratory trials are considered harmless. Pesticides with an effect range from 25% to 49% are considered slightly harmful. Boller *et al*. [27] condensed the first two toxicity classes obtained in field and semi-field tests in one class (N = harmless or slightly harmful), probably to mark the distinction between non-hazardous and hazardous pesticides. Most of the results obtained here fit with records reported in the IOBC Pesticide Side Effect Database while others increase the information available to people involved in IPM strategies.

Chlorpyrifos has been considered harmful for *T*. *pyri* and *A*. *andersoni* in both the laboratory and field [28]. Similar effects are reported for other predatory mites such as *Euseius finlandicus* (Oudemans) (IOBC Pesticide Side Effect Database). Chlorpyrifos has been reported as moderately harmful for *Kampimodromus aberrans* (Oudemans) in field conditions [29]. In other trials chlorpyrifos did not significantly affect predatory mite populations (e.g., [30]). Pesticide history and the appearance of resistance can explain the variability in toxicity encountered in other studies. Barbar *et al*. [31] evaluated the susceptibility to chlorpyrifos by predatory mites (*Typhlodromus exhilaratus* Ragusa) occurring on a vine crop exposed to the pesticide or originating from an unsprayed orchard. As expected, the strain of *T*. *exhilaratus* from the vine was more tolerant to chlorpyrifos as compared to the other strain. Resistance of predatory mites to organophosphates has spread in fruit orchard regions over the world and this provides another explanation for the variability of their impact on target species, including *T*. *pyri* [32]. *K*. *aberrans* strains resistant to chlorpyrifos have been detected in vineyards in Northeastern Italy and mechanisms of resistance have recently been studied [33,34]. The reduction in fecundity of *K*. *aberrans* females exposed to chlorpyrifos can be observed even in resistant strains [35,36]. Such sub-lethal effects have been little explored for other predatory mites. In a field study carried out in North America, chlorpyrifos applications led to higher spider mite densities in vineyards and pesticide impact on generalist phytoseiid mites was probably the key mechanism involved in mite outbreaks [21]. We can conclude that chlorpyrifos should be used with caution in IPM for its negative effects on some predatory mite species. Recent papers have demonstrated that the side effects of chlorpyrifos on predatory mites can be mitigated when they have fresh alternative food sources available [37].

In previous trials (laboratory or field tests) flufenoxuron has been classified as harmless or slightly harmful for *T*. *pyri* (e.g., [2,38]) and slightly harmful for *K*. *aberrans* [29]. In our experiments the impact of flufenoxuron on *T*. *pyri* and especially *A*. *andersoni* appeared to be more deleterious. Variability in the impact of flufenoxuron could depend on species and strain features as well as pesticide history. A laboratory study conducted on two *K*. *aberrans* strains showed low effects of flufenoxuron on the survival of predatory mite females but a significant reduction in the fecundity of females belonging to one of these strains [35].

Thiamethoxam showed some detrimental effects on *T*. *pyri* populations. In other trials where *K*. *aberrans* was involved, effects on survival were negligible to low, while thiamethoxam reduced predatory mite fecundity [35,36]. Similar effects have been reported in studies conducted with other predatory mite species [39,40]. The functional response to prey in predatory mites can be altered by neonicotinoids including thiamethoxam [41]. Moreover, the total effect of thiamethoxam on predatory mites increased with the routes of exposure involved; in particular, residual and contaminated food exposures increased pesticide effects [13]. The use of neonicotinoid insecticides in IPM programs should therefore be carefully evaluated (e.g., [42,43,44]). Little is reported about the susceptibility of predatory mites to tebufenozide. In the IOBC Pesticide Side Effect Database this insecticide is reported as harmless or slightly harmful to *T*. *pyri*. Similar effects have been reported for other predatory mites [45]. Our data refer to *A*. *andersoni* only and confirm the low impact of tebufenozide on predatory mite populations.

Indoxacarb appeared to be the most selective pesticide towards *T*. *pyri* and *A*. *andersoni*, confirming data reported for *K*. *aberrans* [35,36] and other predatory mites [45,46].

The results obtained here provide useful information for the definition of IPM strategies on grapevine. Indoxacarb and tebufenozide had a low impact on the predatory mites considered here, while the highest impact was observed for chlorpyrifos, flufenoxuron, and thiamethoxam. The use of the latter insecticides should be carefully considered in the light of the conservation of predatory mite populations on grapevine.
